# 

**Published:** 1880-04

**Authors:** 


| | PAGE. |
|---|---|
| **Original Communications:** | |
| State Medicine, by Thomas F. Rochester, M. D., | 373 |
| **Clinical Reports:** | |
| Sarcomatous Tumors in the Orbit, by Lucien Howe, M. D., | 385 |
| **Translations:** | |
| J. A. Estlander's Clinical Contributions. Translated from the Swedish by Herman Mynter, M. D., | 389 |
| Puncture of Hæmarthros, by Richard Volkman. Translated from the German by Herman Mynter, M. D., | 393 |
| **Selections:** | |
| Renal Inadequacy, | 394 |
| Post-Partum Hemorrhage, | 397 |
| Amputation of Hip-Joint and Excision, | 402 |
| The Etiology of Puerperal Cystitis, by Swartz, of Halle, A. S., | 403 |
| Miscarriage and Death resulting from Inflammation and Suppuration, | 404 |
| Iodized Phenol—"Battey's Formula"—in Eczema Marginatum, by W. J. H. Bellamy, M. D., | 405 |
| Ovariotomy following Incision and Long-Drainage, | 407 |
| A Case of Nephrotomy, | 407 |
| Conflagration from the use of the Thermo-Cautery during Anæsthesia from Ether, | 407 |
| A New Anthelmintic, | 408 |
| Czerny's Operation for the Radical Cure of Hernia, | 408 |
| Remedy for Night Sweats in Phthisis, | 409 |
| Women as Physicians, | 409 |
| Benzoate of Soda in Diphtheria, | 410 |
| The Treatment of Neuralgia, | 411 |
| Dextro-Quinine in Periodical Hemicrania, | 412 |
| **Society Reports:** | |
| Buffalo Medical Association, | 413 |
| **Editorial:** | |
| Alumni Associations, | 418 |
| **Reviews,** | 420 |

